# Augmentation of GNSS by Low-Cost MEMS IMU, OBD-II, and Digital Altimeter for Improved Positioning in Urban Area

**DOI:** 10.3390/s18113830

**Published:** 2018-11-08

**Authors:** JoonHoo Lim, Won Jae Yoo, La Woo Kim, You Dam Lee, Hyung Keun Lee

**Affiliations:** School of Electronics and Information Engineering, Korea Aerospace University, Goyang-si 10540, Korea; limjh@kau.kr (J.L.); wjyoo@kau.kr (W.J.Y.); lawookim@kau.kr (L.W.K.); leeyd39@kau.kr (Y.D.L.)

**Keywords:** multi-sensor fusion, positioning, time synchronization, urban area

## Abstract

This paper proposes an efficient multi-sensor system to complement GNSS (Global Navigation Satellite System) for improved positioning in urban area. The proposed system augments GNSS by low-cost MEMS IMU (Micro Electro Mechanical Systems Inertial Measurement Unit), OBD (On-Board Diagnostics)-II, and digital altimeter modules. For improved availability of time synchronization in urban area, an adaptive synchronization method is proposed to combine the external PPS (Pulse Per Second) signal and the internal onboard clock. For improved positioning accuracy and availability, a 17-state Kalman filter is formulated for efficient multi-sensor fusion, including OBD-II and digital altimeter modules. A strategy to apply different types of measurement updates is also proposed for improved performance in urban area. Four experiment results with field-collected measurements evaluates the performance of the proposed GNSS/IMU/OBD-II/altimeter system in various aspects, including accuracy, precision, continuity, and availability.

## 1. Introduction

Accurate and continuous positioning is essential for various unmanned aerospace and land vehicles. As widely known, GNSS (Global Navigation Satellite System) is widely used for this purpose nowadays. However, its positioning accuracy and continuity are reduced due to multipath and lack of visible satellites when vehicles are in harsh environments, such as tunnels, underpasses and urban canyons [1,2]. To mitigate the performance degradation, multi-sensor systems to aid or complement GNSS have been studied by many researchers.

One of the most representative research works in this kind is the combination of GNSS and INS (Inertial Navigation System). This combination is quite attractive due to the complementing characteristics of INS and GNSS. However, its error growth rate is highly dependent on INS grade when GNSS signal is not available [1,3]. In the research work by Godha [1], it can be seen that position error can grow up to 15 m in the urban area even after applying an RTS (Rauch-Tung-Striebel) smoothing filter in both forward and backward directions in time. Angrisano proposed an integrated GPS/GLONASS/MEMS IMU (Micro Electro Mechanical Systems Inertial Measurement Unit) system and investigated the effects of using different motion constraints [4]. By experiment results, it was shown that the RMS (Root Mean Square) position error of about 10 m, the velocity error within 1 m/s, and the azimuth error within 10° can be achieved.

To overcome weak points of GNSS/INS have been studied recently. Georgy proposed a particle filter based GPS/MEMS IMU/OBD (On Board Diagnostics) system combining the speed information from the OBD module and the roll and pitch angles estimated by the MEMS IMU [5]. By experiments in urban area, it was shown that the average horizontal position error of about 16 m and the average vertical position error of about 5 m can be obtained. Abdelfatah proposed a 2D-RISS (Reduced Inertial Sensor System) that combines a gyroscope, a GPS receiver, and an OBD-II module [6] by 7-state Kalman filter. By experiment results, it was shown that the RMS horizontal position error was from 6 to 8 m in near open-sky environment with a small number of natural GPS outages. The RMS horizontal position error was from 8 to 16 m after applying four simulated outages of 60 s.

It is well known that vision sensors are effective to prevent the divergence of GNSS/INS. Lanes and landmarks identified by vision sensor can be used to improve positioning accuracy [7,8,9,10]. Vision sensor does not accumulate errors unlike inertial sensors. However, they are easily affected by blocking, blurring, external brightness, weather and so on. To overcome this problem, driving behavior (straight-line driving and lane change) combined with map information was exploited to improve the lateral accuracy of the vehicle [11,12]. Recently, Suhr proposed a localization system for complex urban environment combining GPS, IMU and camera measurements assuming accurate digital map [13]. By experiments in deep urban area, it was shown that mean and standard deviation of Euclidean positioning error were 1.69 m and 1.63 m, respectively.

LiDAR (Light Detection and Ranging) can provide precise range measurements and it is not affected by external brightness and weather. For the reason, it has been widely used for ADAS (Advanced Driver Assistance Systems) [14,15]. LiDAR enables accurate map building regarding lane marks, road signs, speed bumps, crosswalks and so on. The accurate map prepared in advance can aid integrated GNSS/IMU/LiDAR systems for autonomous vehicles [16].

In addition to the utilization of multiple heterogeneous sensors, time synchronization is also an important issue for efficient multi-sensor fusion. Since GPS provides not only position but also time information, it is usually considered as the source for multi-sensor time synchronization. Regarding the time synchronization, three different methods have been studied: Hardware, software, and combined method [17]. The first method corresponds to the hardware-only method that utilizes the PPS (Pulse Per Second) signal from a GPS receiver to trigger multi-sensor sampling directly [18,19,20]. The second method corresponds to the software method that utilizes the Kalman filter [21,22] under a controlled trajectory [23]. The third method maintains an internal counter that is reset by the PPS signal and all the sensor measurements are tagged by the counter value.

For the synchronization of sensor networks, four factors need to be considered. They are transmission time, access time, propagation time, and reception time [24]. For the synchronization of sensor networks, a RBS (Reference Broadcast Synchronization) method was proposed in Ref. [25]. TPSN (Timing-Sync Protocol for Sensor Networks) and system-level optimization method proposed in Refs. [26,27]. The TPSN-based procedure consists of two phases: Discovery and synchronization. In addition, Tiny/Mini-Sync [28] and LTS (Lightweight Tree-Based Synchronization) [29] methods were also studied.

To generate the PPS signal as the reference for time synchronization, several studies have also been carried out. A method utilizing dedicated digital circuit was studied in Ref. [30]. This method reduces jitters in the PPS signal with short settling time. A method to maintain time synchronization accuracy by combining the PPS signal and SNTP (Simple Network Time Protocol) was studied in Ref. [31].

Time synchronization error in integrated GPS/INS causes biased estimation of navigation variables. In particular, abnormal large positioning error can be caused by incorrect accelerometer bias estimation [21]. Later, it was revealed that the incorrect estimation of accelerometer bias is driven by the biased attitude error that is proportional to the time synchronization error [23]. To achieve accurate time synchronization, a method using the DAQ (data acquisition) card was studied [32]. This method considered flexible synchronized data acquisition system.

As briefly overviewed, there have been many studies on multi-sensor fusion to complement the weakness of GNSS in urban area. Most of conventional anti-urban methods utilize high-cost hardware, such as high-accuracy IMU or LiDAR. In many cases, provision of accurate external information (map, land-mark coordinates) is required. However, independence on external prior map information is an essential requirement to build an accurate map autonomously. It is still difficult to obtain continuous and accurate positioning results in urban area without external information.

For improved positioning in urban area, this paper proposes an efficient system that augments GNSS by self-contained sensors without external provision of prior information. The proposed system augments GNSS by low-cost MEMS IMU, OBD-II, and digital altimeter modules. For efficient multi-sensor fusion in GNSS-denied area, adaptive time synchronization is considered. For improved positioning in urban area, a 17-state Kalman filter is formulated for efficient multi-sensor fusion, including OBD-II and digital altimeter modules. In more detail, the contribution of this paper is summarized as follows:

(1) An efficient time synchronization method is proposed to integrate multi-sensors in urban area. The proposed method combines the PPS signal with a low-cost onboard clock adaptively. Thus, time synchronization can be achieved continuously without interrupts in urban area where PPS signal is not available intermittently.
(2) A 17-state Kalman filter is proposed to augment the conventional 15-state loosely-coupled GNSS/INS Kalman filter [19,21,23] by OBD-II and digital altimeter measurements. Since performance degradation of the conventional multi-sensor systems are mainly due to the shortage of GNSS measurements in urban area, the proposed method improves accuracy, continuity, and availability considerably as will be shown by the experiment results.
(3) An efficient strategy to apply different types of measurement updates in urban is proposed. Four types of measurements updates are classified where three types do not depend on GNSS availability.

This paper is organized as follows. At first, an adaptive time synchronization scheme is explained. Next, preparations required to use OBD-II and digital altimeter modules are explained. After this, the 17-state Kalman filter integrating MEMS IMU, GNSS, OBD-II and digital altimeter modules is explained. Finally, experiment results are presented to evaluate the performance of the proposed system in various aspects, including accuracy, precision, continuity, and availability.

## 2. Adaptive Time Synchronization

The simplest time synchronization method for multi-sensor fusion is to utilize GPS messages containing absolute time information [33]. As soon as each message arrives, it is decoded and utilized as the time reference. This method is widely used due to its simplicity. However, this method does not consider the processing time required to decode GPS messages and the propagation time between the transmitter and the receiver.

**Figure 1:**
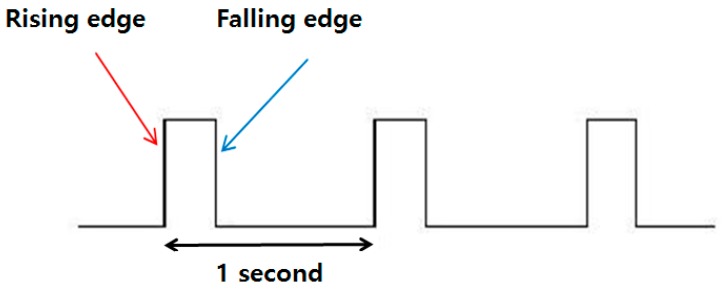
Shape of the pulse per second signal.

A more enhanced method is to utilize the PPS signal instead of GPS messages [19,20]. This method provides more precise time information. Figure 1 shows the shape of the PPS signal. The rising edge of the GPS PPS signal can be easily detected.

**Figure 2:**
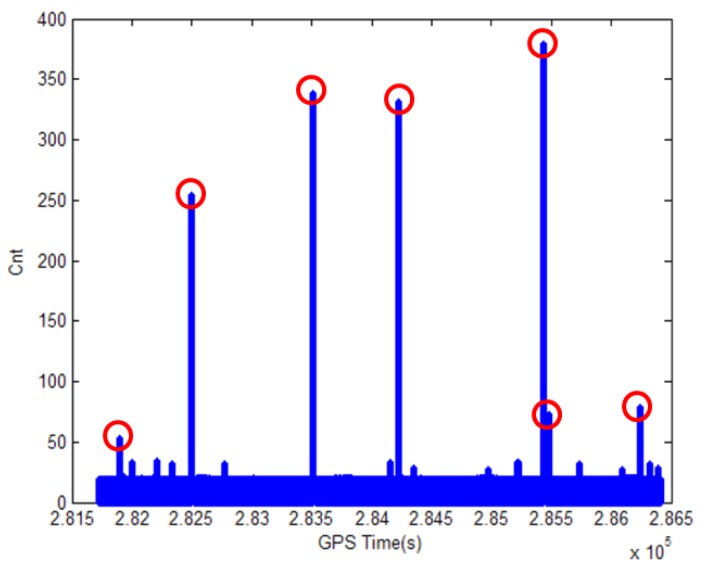
Abnormal counter values due to the loss of GPS pulse per second signal in deep urban area.

However, the PPS signal is not available in GPS-denied area, such as urban canyon, tunnel, or underpass. Figure 2 shows the how the synchronization counter value changes due to the several losses of the PPS signal when the vehicle is moving in deep urban area. In Figure 2, the *x* and *y* axes correspond to the GPS time and the counter value of an embedded board, respectively. The counter value is incremented at $F_{s}$ = 20 Hz rate and reset every second by the PPS signal if it is available. Thus, the value is less than $F_{s}$ in normal operation. However, it can be seen in Figure 2 that there are several cases when the counter value exceeds $F_{s}$ to very large values. The maximum value is observed as 380. This result means that there are several losses of PPS signal and the longest period without the PPS signal is approximately 19 (=380/20) s.

Designing a dedicated digital circuit for time synchronization is too costly in integrating off-the-shelf sensors. In addition, establishing an extra timing server for time synchronization is too complex to synchronize several onboard sensors. Motivated by these shortcomings, this paper proposes an adaptive time synchronization method. The proposed method also extends conventional PPS-based time synchronization methods. However, internal clock of a typical low-cost embedded board assists the PPS signal so that time tagging for multi-sensor synchronization is not interrupted in GNSS-denied area. Thus, the proposed method is a combined hardware and software method, which is flexible to implement on various platforms.

**Figure 3:**
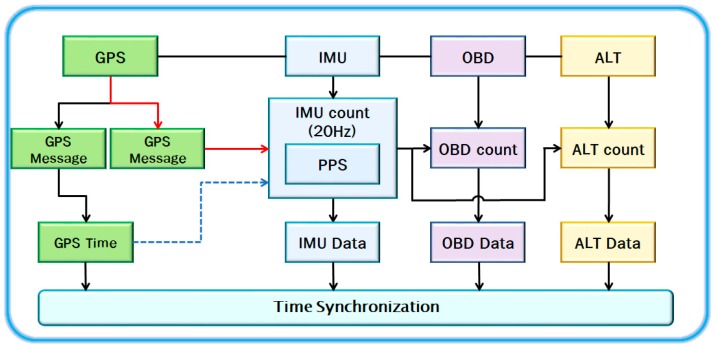
Architecture based on the proposed time synchronization method.

Figure 3 shows an architecture of the synchronization method. The outputs of the GPS receiver, the IMU, the OBD, and the altimeter (printed as ALT in the Figure 3) are sampled at 1 Hz, 20 Hz, 2 Hz, and 1 Hz, respectively. The PPS signal is directly connected to the GPIO (General Purpose Input Output) port to minimize time delay. The rising edge of the PPS signal, if available, is detected to reset the fastest internal counter. In Figure 3, the IMU counter is used as the internal timer, since it is faster than other counters. The IMU timer is divided by 20 and used as the slow altimeter timer. In this way, these timers are utilized to provide the synchronization information in GPS-denied area.

**Figure 4:**
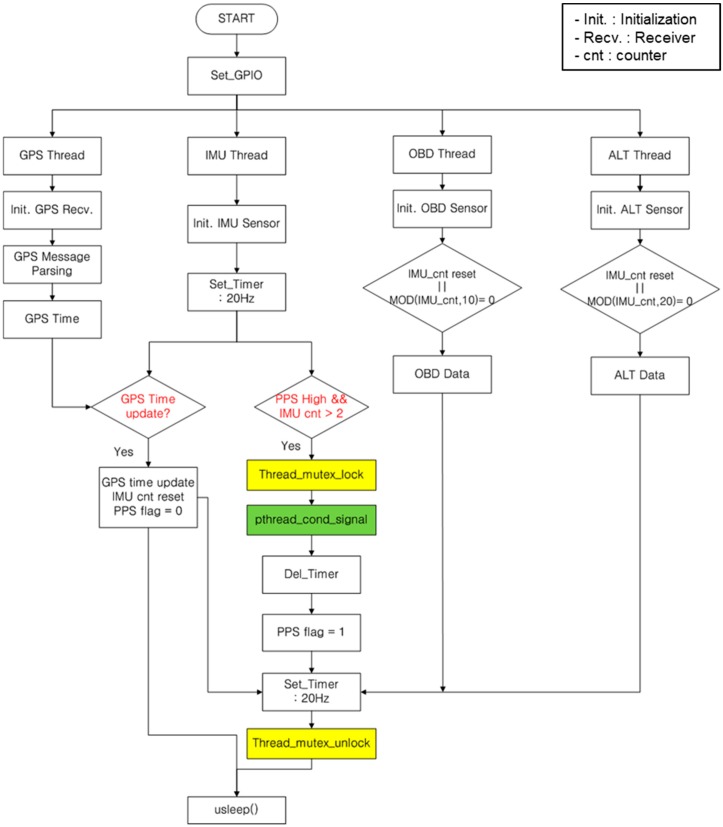
Flow chart of the main algorithm.

The proposed synchronization method consists of two algorithms; main algorithm and timer algorithm. Figure 4 shows the flow chart of the main algorithm. In the proposed method, each sensor module is paired with each independent thread. The GPS thread processes to obtain GPS time information. The IMU thread runs at 20 Hz update rate and monitors the rising edge of the PPS signal. If the rising edge is detected, the IMU count is reset and transmits the initialization signal to OBD and ALT thread. The OBD and ALT thread transmit the speed of the vehicle and altimeter measurements to the IMU thread. To prevent clash of each threads, “Thread_mutex_lock” and “Thread_mutex_unlock” are utilized as protection functions. In addition, “pthread_cond_signal” is used to give priority to each thread.

**Figure 5:**
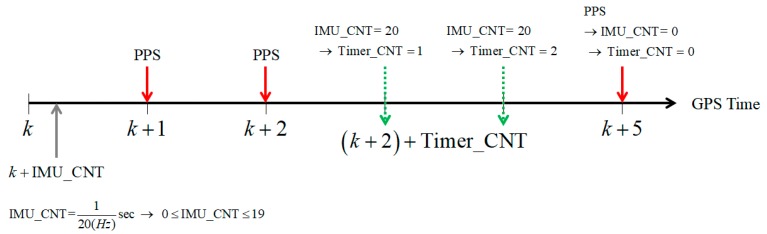
Calculation of the synchronization time.

Figure 5 shows the relationship among the GPS time, the IMU count and the timer count depending on the PPS signal. The GPS time is updated by the PPS signal and the IMU count is dependent on the sampling rate of MEMS sensor. The timer count is updated by the IMU count when the PPS signal is not available. It is only reset by the PPS signal. Therefore, the synchronization time can be calculated by Equation (1) with the GPS time, the IMU count and the timer count.

(1) Synchronization time = GPS time (k) + IMU_CNT + Timer_CNT 

In summary, the proposed method utilizes the GPS PPS signal as the primary source for time synchronization. In addition, the internal clock of the embedded board assists time synchronization when the PPS signal is not available in GPS-denied area.

## 3. Conversion of OBD-II and Digital Altimeter Measurements

The number of visible satellites increases dramatically as we use multiple GNSS constellations instead of single GPS constellation. However, multi-constellation GNSS receivers still suffer from satellite visibility problem in urban area. To alleviate this problem for improved accuracy, continuity, and availability, stable provision of one more measurement is quite beneficial. For this purpose, either a low-cost OBD-II module or a digital altimeter module is very effective in urban area.

An OBD-II module provides diagnostic information of most vehicles according to the international standard [34]. In this paper, the OBD-II module was used to obtain the speed of the vehicle. Based on the PID (Parameter IDs) 0D mode of the OBD-II standard, as shown in Table 1, the speed of vehicle can be obtained [35]. The procedure is summarized by the following steps.

(1) The user sends the PID to the vehicle’s CAN (Controller–Area Network) bus, VPW (Variable Pulse Width), PWM (Pulse-Width Modulation), ISO (International Organization for Standardization), KWP (Keyword Protocol). (After 2008, CAN only);
(2) The processor in the vehicle recognizes the PID and returns the response to the PID;
(3) The scan tool decodes the response message and send it to the user.

The forward velocity obtained by the OBD-II module can be modeled as follows.

(2) $${\hat{V}}_{X}=\left(1+\hat{s}\right){\tilde{u}}_{x}+v_{OBD}\;$$

where,

${\hat{V}}_{x}$: Estimated forward velocity component
${\tilde{u}}_{x}$: Speed measured by OBD-II module
$\hat{s}=s+\delta s$: Estimated scale factor
s: True scale factor
δs: Scale factor estimation error
$v_{OBD}$: OBD speed measurement noise.

**Figure 6:**
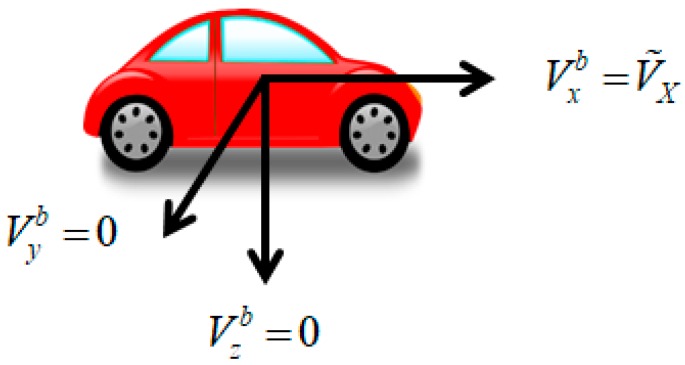
Relationship between the speed and vehicle coordinate system.

Figure 6 shows the configuration of the 3-dimensional velocity components. Among the forward, lateral, and downward directions with respect to the body frame, the speed of a vehicle affects forward direction only. The lateral and downward velocity components are assumed to be zero considering the constraint of vehicle movements. Thus, the 3D velocity vector can be constructed as follows by combining the coordinate transformation matrix provided by the inertial algorithm and the forward speed measurement provided by the OBD-II module.

(3) $${\hat{V}}_{OBD}=\hat{C}\left[\begin{array}{c} {\hat{V}}_{X}\\ 0\\ 0 \end{array}\right]\;$$

where,

${\hat{V}}_{OBD}$: Estimated 3D velocity vector
$\hat{C}$: Estimated transformation matrix from the body frame to the locally-level navigation frame.

**Figure 7:**
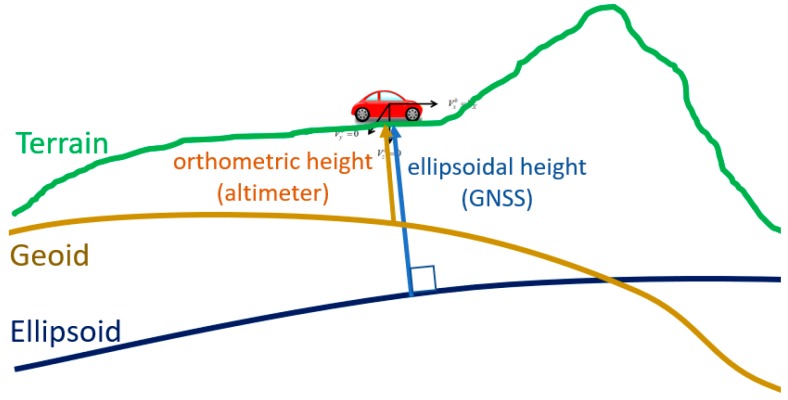
Comparison of ellipsoidal height and orthometric height.

To utilize the digital altimeter, different height standards should be considered. Figure 7 compares two different height standards; ellipsoidal height and orthometric height. The height information provided by a GNSS receiver corresponds to the ellipsoidal height. Measurements provided by a digital altimeter are related to the orthometric height.

To obtain height information from a digital altimeter, the following conversion equation should be applied.

(4) $$H=\frac{273.15+T_{0}}{\Gamma }\times \left\{{\left(\frac{P}{P_{0}}\right)}^{-\Gamma \times \frac{R}{g}}-1\right\}\;(m),$$

where,

H: Height (m)
$T_{0}$: Reference temperature = 15 °C
$P_{0}$: Reference atmospheric pressure = 1013.25 hpa
P: Measured atmospheric pressure
Γ: Temperature decreasing rate = 0.0065 K/m
R: Gas constant = 287.058 $\frac{J}{\mathrm{Kg}\cdot K}$
g: Gravity = 0.980665 ${m/s}^{2}$.

**Figure 8:**
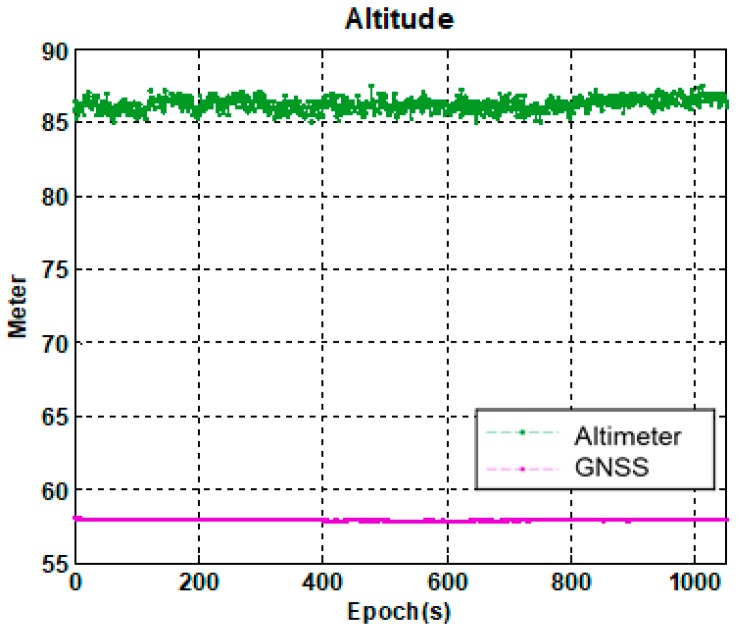
Comparison of height measurements between GNSS and altimeter.

After the conversion by Equation (4), the orthometric height is obtained. Figure 8 shows a typical example that illustrates the difference between the orthometric height obtained by a digital altimeter and the ellipsoidal height obtained by a GNSS receiver. As shown in Figure 8, the difference between the height measurement of the digital altimeter $h_{BARO}$ and the GNSS height $h_{GNSS}$ can be considered as constant d in a small area within short time period.

(5) $$d=h_{GNSS}-h_{BARO},$$

where,

$h_{GNSS}$: Ellipsoidal height obtained by GNSS
$h_{BARO}$: Orthometric height measured by digital altimeter
d: Height difference.

## 4. Kalman Filter for GNSS/IMU/OBD-II/Altimeter Integration

GNSS provides absolute position and time information under globally uniform reference. However, it is prone to multipath error in urban area. Under severe environments, such as tunnels and underpasses, it cannot provide position and time information at all. An IMU processed by strapdown inertial algorithm can provide incremental position, velocity and attitude. With high-quality IMUs, the high-precision inertial algorithm can supplement GNSS in urban area during considerable time interval. However, with low-cost MEMS IMUs, positioning error growth cannot be bounded. For the reason, the integration of GNSS and low-cost MEMS IMU cannot provide satisfactory positioning accuracy in urban area.

An OBD-II module provides the speed information that is equivalent to the incremental position in the forward direction. Thus, it is less affected by the error growth compared with the MEMS IMU which provides incremental velocity. A digital altimeter can provide absolute height information when it is properly calibrated. Thus, unlike the inertial height that is the double integral of vertical acceleration, its error magnitude can be bounded during considerable time interval.

Considering the characteristics of different sensors explained so far, a sensor fusion algorithm based on a 17-state Kalman filter can be formulated. Since the self-contained MEMS IMU provides measurements at the highest sampling rate, it takes the central role in the proposed multi-sensor fusion algorithm. The 17-state Kalman filter deals with the following state vector.

(6) $$X={\left[\begin{array}{ccc} \delta X_{INS} & \delta s & \delta d \end{array}\right]}_{}^{T},$$

where,

$\delta X_{INS}={\left[\begin{array}{ccccc} \delta X_{POS} & \delta V & \psi & \nabla & \varepsilon \end{array}\right]}^{T}$
$\delta X_{POS}={\left[\begin{array}{ccc} \delta L & \delta l & \delta h \end{array}\right]}^{T}$: Latitude, longitude, and height errors
$\delta V={\left[\begin{array}{ccc} \delta V_{N} & \delta V_{E} & \delta V_{D} \end{array}\right]}^{T}$: Velocity error
$\psi ={\left[\begin{array}{ccc} \psi_{N} & \psi_{E} & \psi_{D} \end{array}\right]}^{T}$: Attitude error
$\nabla ={\left[\begin{array}{ccc} \nabla_{X} & \nabla_{Y} & \nabla_{Z} \end{array}\right]}^{T}$: Accelerometer bias
$\varepsilon ={\left[\begin{array}{ccc} \varepsilon_{X} & \varepsilon_{Y} & \varepsilon_{Z} \end{array}\right]}^{T}$: Gyro bias
δs: OBD-II scale factor error
δd: Height difference error.

Kalman filter operation consists of two steps; time propagation and measurement update. The time propagation step is based on the system dynamics model summarized in Equation (7).

(7) $$X_{k+1}=F_{k}^{}X_{k}^{}+W_{k}^{},$$

where,

$F_{k}={\left[\begin{array}{ccc} F_{INS} & 0_{15\times 1} & 0_{15\times 1}\\ 0_{1\times 15} & 0 & 0\\ 0_{1\times 15} & 0 & 0 \end{array}\right]}_{17\times 17}$
$F_{INS}$: System matrix representing 15-state INS error dynamics [19,21,23,36]
$W_{k}^{}$: Process noise determined by inertial sensor error characteristics.

**Figure 9:**
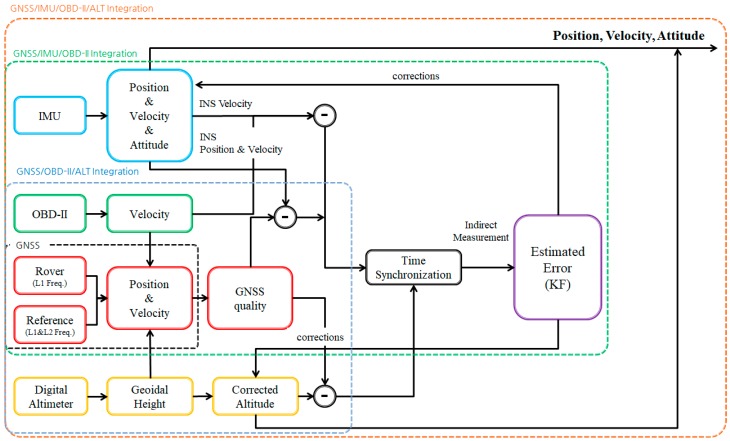
Algorithm of proposed sensor fusion positioning method.

The overall architecture of the proposed method is summarized in Figure 9. As shown in Figure 9, navigation variables, including position, velocity, and attitude are computed by processing IMU outputs at high rate. Then, the inertial navigation variables are compared with the other sensors’ outputs at lower sampling rates. By performing a measurement update among several different types, the Kalman filter corrects the navigation variables.

The difference between the inertial velocity and the OBD-II velocity is modeled by the following equation.

(8) $$\begin{array}{ll} {\hat{V}}_{INS}-{\hat{V}}_{OBD} & =V_{}+\delta V_{INS}-\left\{\left(C_{b}^{n}+\delta C_{b}^{n}\right)\left[\begin{array}{c} (1+k+\delta k){\tilde{u}}_{x}\\ 0\\ 0 \end{array}\right]+v_{OBD}\right\}\\ & =\delta V_{INS}-{\hat{V}}_{OBD}\times \psi -{\hat{C}}_{1}{\tilde{u}}_{x}\delta k-v_{OBD} \end{array}$$

where,

${\hat{C}}_{b}^{n}=C_{b}^{n}+\delta C_{b}^{n}=\left[\begin{array}{ccc} C_{1} & C_{2} & C_{3} \end{array}\right]$
${\hat{C}}_{1}$: First column of ${\hat{C}}_{b}^{n}$
$v_{OBD}$: OBD-II measurement noise.

The difference between the estimated height and the GNSS height is modeled by the following equation.

(9) $$\begin{array}{ll} {\hat{h}}_{ALT}-{\tilde{h}}_{GNSS} & =h_{BARO}+d+\delta d+v_{BARO}-h_{GNSS}-v_{GALT}\\ & =\delta d+v_{BARO}-v_{GALT} \end{array}$$

where,

${\hat{h}}_{ALT}={\tilde{h}}_{BARO}+\hat{d}$
$\hat{d}=d+\delta d$: Estimated height difference
δd: Height difference estimation error
$v_{BARO}$: Altimeter measurement noise
$v_{GALT}$: GNSS height error.

Based on the modeling by Equations (6) and (9), the following equation can be formulated for measurement updates when a sufficient number of GNSS satellites are visible.

(10) $$Z={\left[\begin{array}{c} {\hat{P}}_{INS}-{\tilde{P}}_{GNSS}\\ {\hat{V}}_{INS}-{\tilde{V}}_{GNSS}\\ {\hat{V}}_{INS}-{\hat{V}}_{OBD}\\ {\hat{h}}_{ALT}-{\tilde{h}}_{GNSS} \end{array}\right]}_{10x1}=H\hat{X}-{\left[\begin{array}{c} v_{GPOS}\\ v_{GVEL}\\ v_{OBD}\\ v_{GALT}-v_{BARO} \end{array}\right]}_{10x1}\;$$

where,

$H={\left[\begin{array}{ccccccc} I_{3x3} & 0_{3x3} & 0_{3x3} & 0_{3x3} & 0_{3x3} & 0_{3x1} & 0_{3x1}\\ 0_{3x3} & I_{3x3} & 0_{3x3} & 0_{3x3} & 0_{3x3} & 0_{3x1} & 0_{3x1}\\ 0_{3x3} & I_{3x3} & -\left({\hat{V}}_{OBD}\times \right) & 0_{3x3} & 0_{3x3} & -{\hat{C}}_{1}{\tilde{u}}_{x} & 0_{3x1}\\ 0_{1x1} & 0_{1x1} & 0_{1x1} & 0_{1x1} & 0_{1x1} & 0_{1x1} & 1 \end{array}\right]}_{10\times 17}$
(y×): 3×3 skew-symmetric matrix constructed by 3×1 vector y.

In GNSS-denied environments, including urban canyon, tunnel, or underpass where GNSS position and velocity are not available, the following equation can be utilized for measurement updates.

(11) $$Z={\left[\begin{array}{c} {\hat{V}}_{INS}-{\hat{V}}_{OBD}\\ {\hat{h}}_{alt}-{\tilde{h}}_{GNSS} \end{array}\right]}_{4x1}=H\hat{X}-{\left[\begin{array}{c} v_{OBD}\\ v_{GALT}-v_{BARO} \end{array}\right]}_{4x1},$$

where,

$H={\left[\begin{array}{ccccccc} I_{3x3} & 0_{3x3} & 0_{3x3} & 0_{3x3} & 0_{3x3} & 0_{3x1} & 0_{3x1}\\ 0_{3x3} & I_{3x3} & -\left({\hat{V}}_{OBD}\times \right) & 0_{3x3} & 0_{3x3} & -{\hat{C}}_{1}{\tilde{u}}_{x} & 0_{3x1}\\ 0_{1x1} & 0_{1x1} & 0_{1x1} & 0_{1x1} & 0_{1x1} & 0_{1x1} & 1 \end{array}\right]}_{7\times 17}$.

In urban area, vehicles frequently stop to wait for traffic signs. In these cases, linear and angular velocities become zero apparently. The zero linear and angular velocity conditions can be used for measurement updates as follows. This type of measurement update is quite useful in inertial sensor bias calibration.

(12) $$Z={\left[\begin{array}{c} {\hat{V}}_{INS}\\ {\tilde{\omega }}_{GYRO} \end{array}\right]}_{6\times 1}=H\hat{X}+\left[\begin{array}{c} v_{ZVEL}\\ v_{GYRO} \end{array}\right],$$

where,

${\tilde{\omega }}_{GYRO}$: Angular rate measured by gyro
$H={\left[\begin{array}{ccccccc} 0_{3x3} & I_{3x3} & 0_{3x3} & 0_{3x3} & 0_{3x3} & 0_{3x1} & 0_{3x1}\\ 0_{3x3} & 0_{3x3} & 0_{3x3} & 0_{3x3} & I_{3x3} & 0_{3x1} & 0_{3x1} \end{array}\right]}_{6\times 17}$.

Table 2 summarizes the four types of measurement updates explained so far. As shown in Table 2, the proposed method performs the Kalman filter measurement updates regardless of the GNSS satellite visibility. The proposed method corrects the velocity error continuously by utilizing zero velocity or OBD-II velocity. In addition, the height error is always compensated by the digital altimeter. For the reason, the rapid growth of position errors during partial or no satellite visibility can be bounded even if low cost MEMS IMUs are used.

## 5. Experiment

To evaluate the performance of the proposed method, four experiments were performed. The first experiment was performed to compare the accuracy of the proposed method with the vision sensor aided positioning method in a relatively open area. The second experiment was performed to evaluate the continuity and precision of the proposed method in typical urban areas. The third experiment was purposed to evaluate the validity of the proposed adaptive synchronization method in urban area. The final experiment was purposed to evaluate the accuracy of the proposed method under simulated signal blockages imitating urban area.

**Figure 10:**
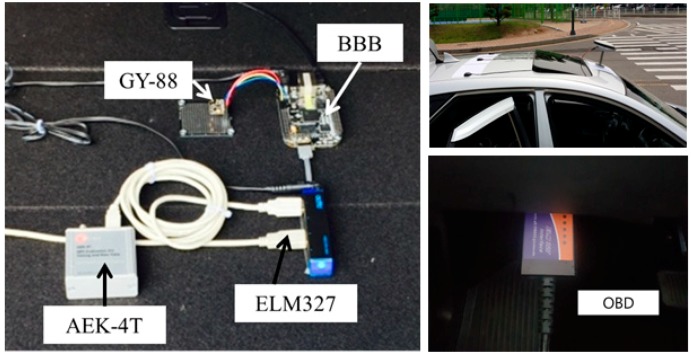
Sensor locations used in the experiments.

Table 3 summarizes the sensors used in the experiment. The RMS noise of gyro and accelerometer of MPU-6050 module are 0.05°/s, 400 $\mu g/\sqrt{\mathrm{Hz}}$ respectively. It is so large that inertial coasting without GNSS signal is practically difficult even during several seconds. Figure 10 shows the locations of the sensors used in the experiments.

### 5.1. Comparison with Vision-Aided Positioning Method in Open Area

**Figure 11:**
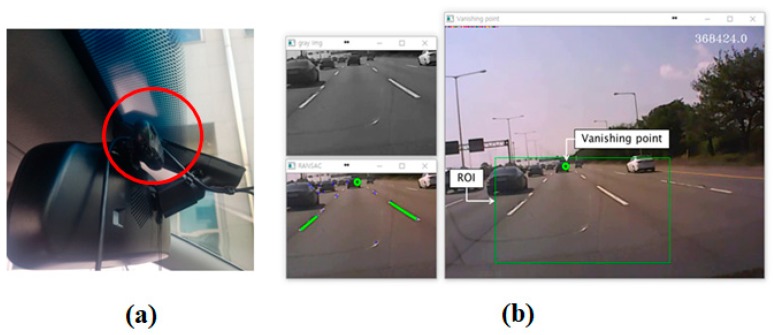
(**a**) Vision sensor; Logitech C170; (**b**) detected lanes and vanishing point.

**Figure 12:**
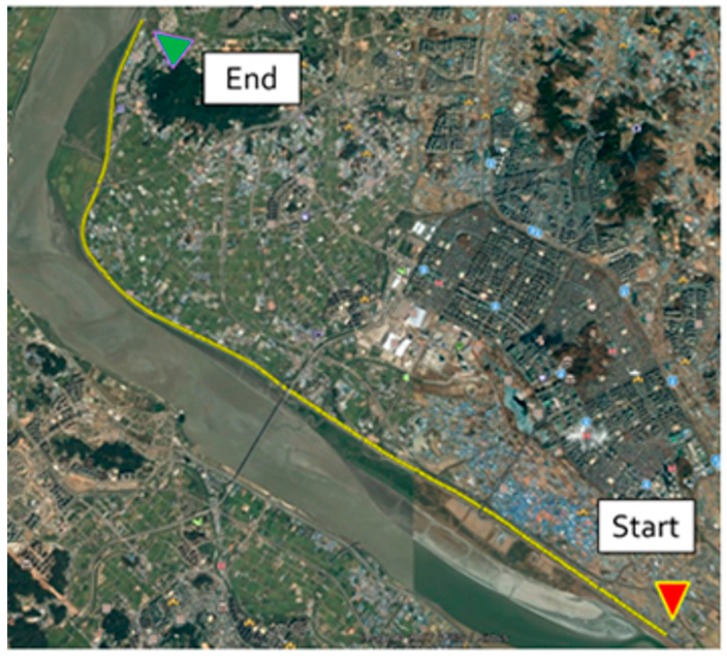
Experimental environments and trajectories; GNSS_S; GNSS_D; GNSS_V; GNSS_P.

The first experiment is purposed to compare the proposed method (GNSS_P) with the vision sensor aided positioning method (GNSS_V) in a relatively open area. Figure 11a shows the vision sensor installed in the vehicle. Figure 12 shows the experiment area and the trajectory. The GNSS_V method utilizes the straight movement of the vehicle to improve the position accuracy. The vanishing points of straight road lanes are utilized to estimate the heading of the vehicle relative to the road lane [7].

Since the experiment area provides sufficiently many visible satellites, the dual frequency RTK (Real-Time Kinematic) method can provide cm-level accuracy based on integer ambiguity resolution. For the reason, the RTK trajectory was used as the truth data for accuracy statistics.

The RTK method depends only on GNSS pseudorange and carrier phase measurements. It resolves integer ambiguities in carrier phase measurements. To prepare for the integer ambiguity resolution, pseudorange measurements are smoothed by carrier phase measurements during a fixed time interval. With the smoothed pseudorange measurements, a float solution is generated as a marginally accurate estimate of receiver position. Based on the float solution, candidates of integer ambiguities contained in carrier phase measurements are generated. By applying a verification procedure, optimal integer ambiguities are resolved. With the resolved integer ambiguities, an integer solution can be generated as an accurate estimate of receiver position.

**Figure 13:**
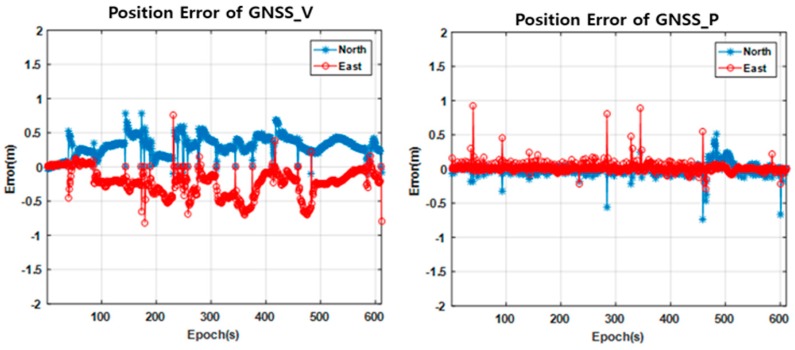
Comparison of positioning error profiles of the vision sensor aided positioning method (GNSS_V) and the proposed method (GNSS_P).

Figure 13 and Table 4 compare the positioning errors of the GNSS_V and GNSS_P methods with respect to the trajectory generated by the dual frequency RTK method. In both plots of Figure 13, it can be seen that several discrete jumps occur at the same time. These jumps occur due to the changes in visible satellites. It can also be seen that the jump magnitudes are different by the two methods. By Figure 13 and Table 4, it can be seen that the GNSS_P method shows significantly more accurate trajectory than the GNSS_V methods in terms of RMSE (root mean square error).

### 5.2. Evaluation of Continuity and Precision in Urban Area

**Figure 14:**
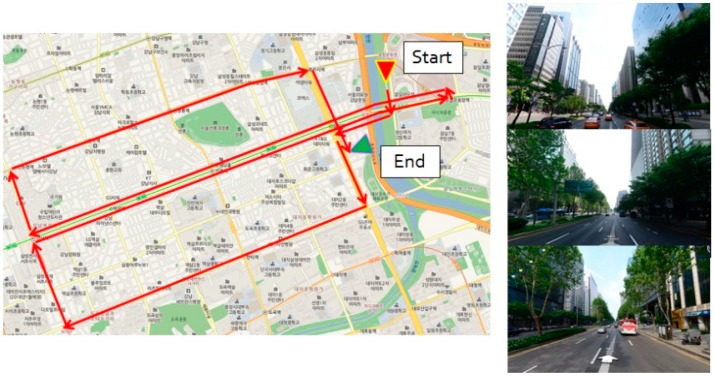
Urban experiment area: Teheran-ro, Gangnam-gu, Seoul, Republic of Korea.

The second experiment is purposed to evaluate the continuity and precision of the proposed method in urban area. Figure 14 shows the trajectory and three representative appearances of the experiment environment in Teheran-ro, Gangnam-gu, Seoul, Republic of Korea. This area corresponds to a typical deep urban environment where navigation signals are blocked frequently due to skyscrapers, street trees, buses, and other multipath errors. Due to the environmental characteristics, no accurate reference data is available for this experiment.

**Figure 15:**
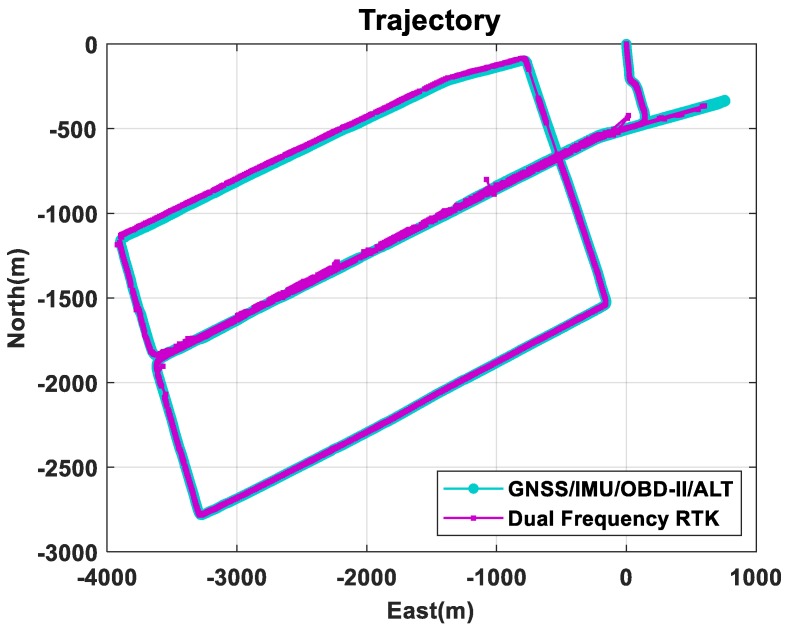
Trajectory of the urban area experiment.

**Figure 16:**
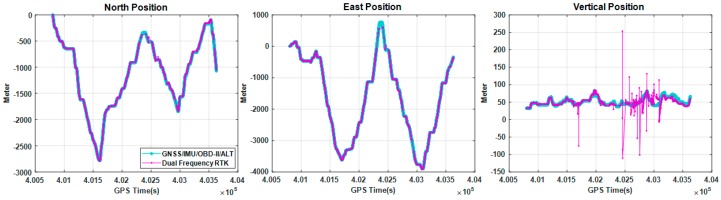
Comparison of variations of north, east, and vertical positions estimated by the proposed method and the conventional dual frequency real-time kinematic method.

Figure 15 compares the trajectories of the proposed method and the conventional RTK method [41]. The proposed method utilized L1 frequency measurements, but the conventional RTK software utilized all the L1 and L2 frequency measurements. The RTK trajectory includes both float and integer solutions, since the availability of the integer-only solutions is very poor in this experiment. Figure 16 compares the variations of north, east, and vertical positions, respectively. As shown in this figure, the proposed multi-sensor fusion system utilizing single frequency measurements generates more continuous positions than the dual frequency RTK method.

**Figure 17:**
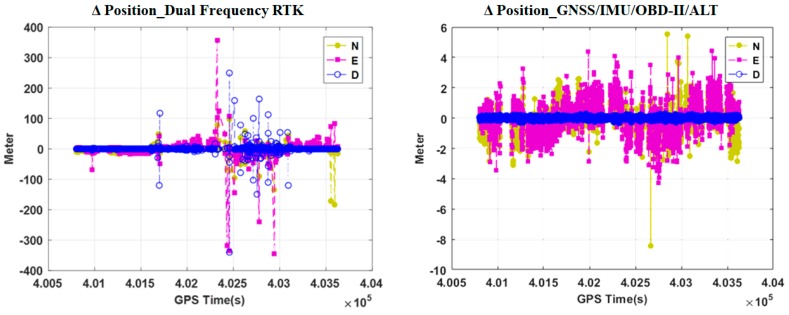
Trends of delta positions of the proposed method and the conventional dual frequency RTK method.

Figure 17 shows the variations of instantaneous delta positions computed by the following formula. The delta position was calculated by the difference between the current and previous position estimates.

(13) ΔPOS(k)=POS(k)−POS(k−1).

Table 4 summarizes the RMS values of the instantaneous delta positions in the north, east, and the vertical directions, respectively. By both Figure 17 and Table 5, it can be seen that the proposed method generates more continuous and precise trajectory than the dual frequency RTK method. It can also be seen the improvement of continuity and precision is significantly apparent due to the utilization of OBD-II and altimeter modules. As explained previously, accuracy cannot be evaluated in this experiment, since accuracy and precision are different performance criteria. Accuracy will be evaluated in the next experiments.

### 5.3. Evaluation of Time Synchronization Accuracy and Availbility in Urban Area

To overcome the limitation of the conventional PPS-based time synchronization methods, the proposed adaptive time synchronization method utilizes the GPS PPS signal as the primary synchronization signal and uses the internal clock of an embedded board to compensate the asynchronous problem in GPS-denied area.

**Figure 18:**
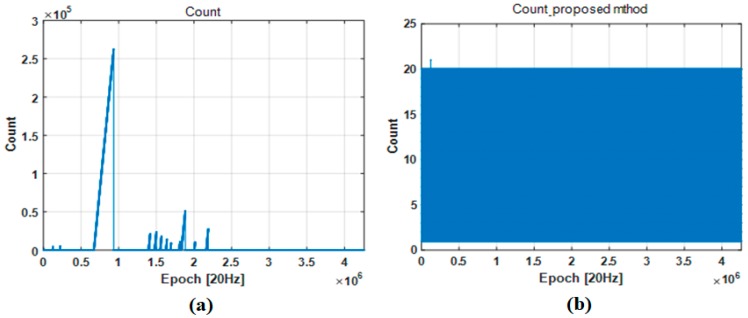
Logging data to validate keeping synchronization: (**a**) counter value of IMU without proposed method; (**b**) counter value of IMU with proposed method.

Figure 18 and Table 6 show the experiment results comparing the maximum counter values without GPS PPS signal availability. These results show how stably the proposed method can maintain the onboard counter with or without the PPS signal. By comparing of Figure 18a,b, it can be confirmed that the maximum counter value is kept within 20 except a single epoch during 60.3 h of experiment by the proposed method. However, the maximum counter value increases largely in several cases without the proposed method. As shown in Figure 18a and Table 6, the onboard fractional counter reaches 262,818, which means that the PPS signal is not available for 262,818/20 s.

**Figure 19:**
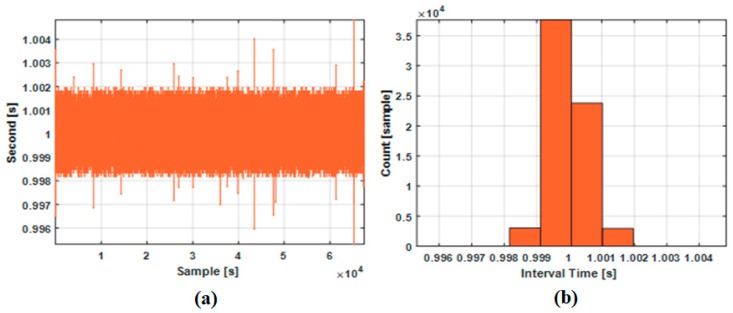
Time intervals between two synchronized epochs: (**a**) trend; (**b**) histogram.

The second part of this experiment is to evaluate the time synchronization accuracy when the PPS signal is always available. Figure 19 shows the incremental time interval Δt(k) defined by the following equation.

(14) $$\Delta t(k)=t_{PPS}(k)-t_{PPS}(k-1)\;$$

Figure 19a shows the trend of incremental time intervals measured between two synchronized epochs. When the PPS signal was generated exactly in time and the interval counter detected the signal immediately, the incremental time interval would be 1.0 s ideally without measurement error. Thus, deviations from 1.0 s in Figure 19a shows how accurate the proposed time synchronization is. Figure 19b shows the histogram of the incremental measured time intervals.

(15) $$\Delta t_{ACCUM}(k)=\sum_{i=0}^{k}\left(\Delta t(i)-1.0\right).$$

Table 7 summarizes the results of this experiment. In Table 7, the most important accuracy parameter is the RMSE of the time synchronization error. As shown in the third column, the proposed method shows the RMSE less than 0.5 millisecond. As shown in the fourth column, the difference between the maximum time interval and the reference time interval of 1.0 s was less than 5 milliseconds. Thus, the time synchronization error does not exceed the shortest sensor output period of 50 milliseconds, which corresponds to 20 Hz sampling rate.

### 5.4. Evaluation of Positioning Accuracy by Simulated Urban Environment

**Figure 20:**
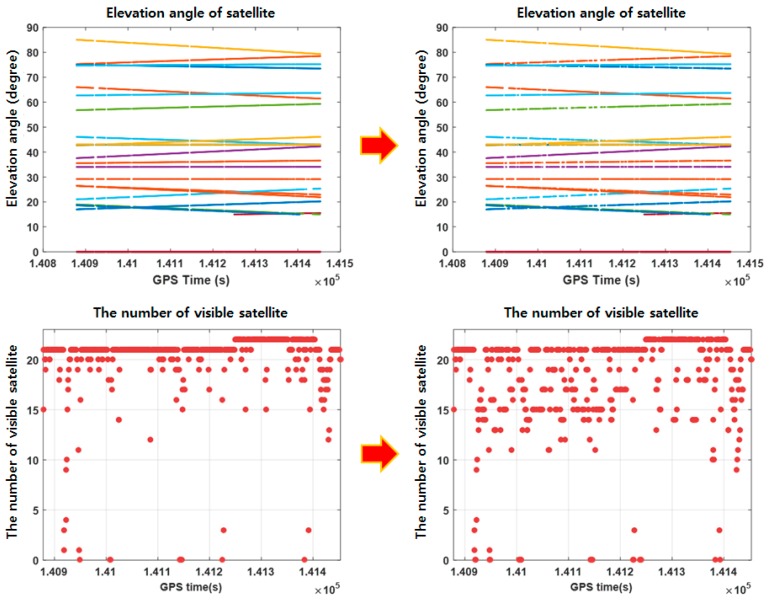
The number of visible satellites simulated for accuracy assessment.

The last experiment is purposed to overcome the difficulty in generating accurate reference trajectory in urban area. For the purpose, sensor measurements were collected in a marginally open-sky area. Next, accurate reference trajectory was generated by the cm-level RTK integer solutions. After this, parts of visible satellites were blocked simulating urban area. Figure 20 shows how the visible satellites were intentionally reduced in this experiment simulating urban area. The signals from satellites at the elevation angle between 15° and 50° are blocked. Table 8 summarizes the conditions used in simulating urban area.

**Figure 21:**
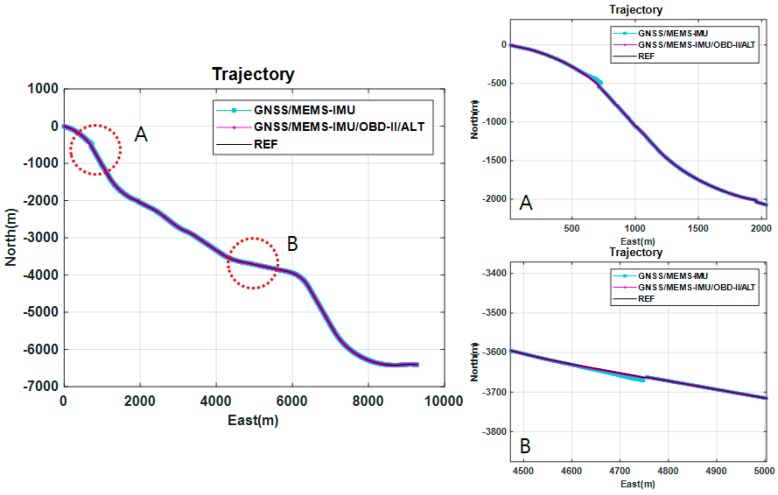
Trajectory of experiment: Reference, GNSS/MEMS IMU, GNSS/MEMS IMU/OBD-II/ALT.

Figure 21 compares the trajectories of the proposed method and the conventional GNSS/MEMS IMU method that utilizes the conventional 15-state Kalman filter. In this figure, two areas are marked by A and B. They represent tunnels. By comparing the two trajectories, it can be observed that the proposed method shows less jumps than the conventional method as soon as the vehicle leaves the tunnels. Table 9 compares the position errors of the GNSS-only method, the integrated GNSS/MEMS IMU method, and the proposed method under simulated satellite signal blockages. As shown in Table, the GNSS/IMU method is four times more accurate than the GNSS-method. The GNSS-only method cannot provide positioning results in tunnels. It is also shown that the proposed method can provide position estimates with the RMSE of 0.67, 0.56, and 0.48 m in the north, east, and vertical directions, respectively. Thus, the RMSE of the proposed method is three times more accurate than the GNSS/IMU method in terms of RMS errors.

### 5.5. Results of the Experiments

This subsection summarizes the results of the four experiments. The first experiment was performed to compare the accuracy of the proposed method with the vision sensor aided positioning method in a relatively open area. By the experiment result, it was shown that the proposed method generates cm-level RMSE. It was also shown that the proposed method is significantly more accurate than the vision sensor aided positioning method in open area where satellite visibility is good.

The second experiment was performed to check the continuity and precision of the proposed method in urban area. Due to the environmental characteristics, no reference data was available for accuracy error statistics. For the reason, RMS of delta positions was used instead of RMSE as the performance measure to compare the precision and continuity of the proposed method and the dual frequency RTK method. It was shown that the proposed method is significantly more advantageous in terms of continuity and precision in urban area.

The third experiment was purposed to evaluate the accuracy and availability of the proposed adaptive time synchronization method in urban area. It was shown that the proposed method can achieve multi-sensor time synchronization with the RMSE less than 0.5 millisecond even when PPS signal is blocked during 3.4764 h.

The final experiment was purposed to evaluate the accuracy of the proposed method under simulated signal blockages imitating urban area. This experiment compared the proposed method based on the 17-state Kalman filter with the GNSS/IMU method based on the conventional 15-state Kalman filter. It was shown that the proposed method is three times more accurate than the conventional GNSS/IMU method in terms of RMSE under simulated urban environment.

## 6. Conclusions

This paper proposed a low-cost multi-sensor system for improved positioning in urban area. The proposed system integrates GNSS, MEMS IMU, OBD-II and digital altimeter. For efficient multi-sensor fusion, an adaptive time synchronization method, a 17-state Kalman filter, and a strategy to apply different types of measurement updates were proposed. To evaluate the performance of the proposed system, four different experiments were performed with field-collected data. By the experiment results, it was shown that the proposed method can improve accuracy, precision, continuity, and availability of position solutions in urban area where GNSS satellites are not sufficiently visible.

## Figures and Tables

**Table 1:** OBD-II mode to obtain vehicle speed [35].

PID (hex)	Data Bytes Returned	Description	Min Value	Max Value	Units
0D	1	Vehicle speed	0	255	km/h

**Table 2:** Classification of measurement update types.

Update Type	GNSS Availability	Vehicle Condition	Related Variable	Measurement Update State
1	O	Moving	position, velocity	- GPS position, velocity
2	O/X	Moving	Velocity	- OBD-II velocity
3	O/X	Moving	Height	- Digital altimeter
4	O/X	Stop	velocity	- Zero linear velocity - Zero angular velocity

**Table 3:** Sensors used in the experiment.

Sensor	Product		Communication Type	Target	Sampling Rate
Embedded Linux board	BeagleBone Black (BBB) [37]		USB	PC	20 Hz
GNSS receiver	u-Blox AEK-4T Novatel ProPak6		USB	PC	1 Hz
			USB	PC	1 Hz
MEMS IMU	GY-88	MPU-6050 [38]	I2C	BBB	20 Hz
MEMS ALT		BMP-085 [39]	I2C	BBB	1 Hz
OBD-II	ELM-327 [40]		USB	BBB	2 Hz

**Table 4:** Comparison of root mean square errors of the vision sensor aided positioning method (GNSS_V) and the proposed method (GNSS_P).

Method	RMSE (m)	
	North	East
Vision sensor aided positioning method (GNSS_V)	0.33084	0.29500
Proposed method (GNSS_P)	0.08750	0.09086

**Table 5:** Comparison of root mean square values of delta positions of the proposed method and the dual frequency RTK (Real-Time Kinematic) method.

RMS_∆POS (m)	N	E	D
Dual Frequency RTK	10.24	20.01	13.97
GNSS/IMU/OBD-II/ALT	0.28	0.45	0.03

**Table 6:** Maximum counter values with or without proposed method.

Total Experiment Time	Max “Count”		Max Time Interval Without PPS Signal
	Without Proposed	With Proposed	
216,108 s (60.03 h)	262,818	21	12,515 s (3.4764 h)

**Table 7:** Results of the accuracy test experiment.

Total Experiment Time	Mean Time Interval (s)	RMSE (s)	Max. Time Interval (s)
67,440 s (19.73 h)	1.0000509171	0.0004632891	1.004823

**Table 8:** Urban simulation conditions.

	Mean (# Visible Satellites)	# Visible Satellites == 0	# Visible Satellites < 5
Original data	20.10	10 s	16 s
Simulated data	17.91	24 s	30 s

**Table 9:** Comparison positioning error of the GNSS-only method, the integrated GNSS/MEMS IMU method, and the proposed method under simulated visible satellite blockages. Bold notation designates the parameters and values.

Method		North (m)	East (m)	Vertical (m)
GNSS	Max	53.495	41.992	22.062
	Min	−29.857	−67.260	−12.296
	Mean	3.298	−4.974	0.365
	**RMSE**	**9.184**	**12.927**	**4.568**
GNSS/MEMS IMU	Max	12.119	8.621	15.884
	Min	−18.795	−25.612	−1.503
	Mean	−0.090	−0.231	0.225
	**RMSE**	**2.173**	**2.457**	**0.957**
Proposed	Max	4.312	6.735	7.942
	Min	−7.907	−5.923	−0.749
	Mean	−0.011	0.055	0.112
	**RMSE**	**0.671**	**0.562**	**0.478**
